# Detecting and diagnosing hotspots for the enhanced management of hospital emergency departments in Queensland, Australia

**DOI:** 10.1186/1472-6947-13-132

**Published:** 2013-12-05

**Authors:** Sarah Bolt, Ross Sparks

**Affiliations:** 1 CSIRO Computational Informatics, Locked Bag 17, 1670 North Ryde NSW, Australia

**Keywords:** Outbreak detection, Disease surveillance, Multivariate control charts, Emergency departments, EWMA control chart

## Abstract

**Background:**

Predictive tools are already being implemented to assist in Emergency Department bed management by forecasting the expected total volume of patients. Yet these tools are unable to detect and diagnose when estimates fall short. Early detection of hotspots, that is subpopulations of patients presenting in unusually high numbers, would help authorities to manage limited health resources and communicate effectively about emerging risks. We evaluate an anomaly detection tool that signals when, and in what way Emergency Departments in 18 hospitals across the state of Queensland, Australia, are significantly exceeding their forecasted patient volumes.

**Methods:**

The tool in question is an adaptation of the Surveillance Tree methodology initially proposed in Sparks and Okugami (IntStatl 1:2–24, 2010). for the monitoring of vehicle crashes. The methodology was trained on presentations to 18 Emergency Departments across Queensland over the period 2006 to 2008. Artificial increases were added to simulated, in-control counts for these data to evaluate the tool’s sensitivity, timeliness and diagnostic capability. The results were compared with those from a univariate control chart. The tool was then applied to data from 2009, the year of the H1N1 (or ‘Swine Flu’) pandemic.

**Results:**

The Surveillance Tree method was found to be at least as effective as a univariate, exponentially weighted moving average (EWMA) control chart when increases occurred in a subgroup of the monitored population. The method has advantages over the univariate control chart in that it allows for the monitoring of multiple disease groups while still allowing control of the overall false alarm rate. It is also able to detect changes in the makeup of the Emergency Department presentations, even when the total count remains unchanged. Furthermore, the Surveillance Tree method provides diagnostic information useful for service improvements or disease management.

**Conclusions:**

Multivariate surveillance provides a useful tool in the management of hospital Emergency Departments by not only efficiently detecting unusually high numbers of presentations, but by providing information about which groups of patients are causing the increase.

## Background

Every year hospital Emergency Departments (EDs) around the world come under increasing pressure as the demands on their resources increase [1-3]. This pressure can reach a critical point in winter when the effects of influenza and other respiratory problems cause EDs to become overcrowded and access to inpatient beds in the rest of the hospital becomes blocked [4,5]. Together these problems are acknowledged to contribute to poorer patient outcomes [6], increased mortality [7], and can result in the cancellation of elective surgeries and the consequent lengthening of waiting lists.

These issues have affected hospitals in the Australian state of Queensland. Queensland has seen a steady increase in influenza cases presenting to EDs [8] and a renewed focus on improving efficiencies in patient access to treatment [9]. These pressures have led to the development and implementation of the PAPT tool to predict ED presentations [10]. The PAPT tool assists managers in planning bed allocations and scheduling resources.

However, many factors contribute to the incidence of winter disease outbreaks, so most predictive tools will inevitably fall short at some point. In these cases, an additional prospective surveillance tool could alert managers to a change in the process underlying the number of patient presentations by signalling a departure from the expected presentation counts. Furthermore, a multivariate surveillance tool could potentially identify which types of patients contibute to this departure. Managers would then have the information required to make short-term changes in resource allocations or apply other management initiatives. For example, a new strain of flu might hit the state and affect a particular age group, e.g. pre-schoolers, more dramatically. Early identification of this group would allow for a shift in paediatric resources, as well as the possibility of targeted public interventions/awareness campaigns or school closures.

There is copious Statistical Process Control literature on the use of univariate techniques to monitor for unusual increases in the incidence of disease; see [11-13] for a selection of applications. However, monitoring a single, aggregated group of patients is likely to be inefficient if the increase occurs only in one subgroup. But, on the other hand, Unkel et.al. [11] pointed out that since the behaviour of subgroups is likely to be correlated, simple multiple application of univariate methods to each of many subgroups would be similarly inefficient. The latter method would also fail to control the overall false alarm rate of the surveillance. If we can detect groups whose behaviour changes together then their needs can be managed jointly or an intervention can be targeted effectively.

The need for combined monitoring of multiple streams of evidence has led to increased interest in multivariate disease surveillance techniques. To date, most methods find hotspots by essentially performing exhaustive searches in the target space. For example, extension of the popular spatio-temporal SCAN statistic by Kulldorf defines a test statistic that incorporates an adjustment for multiple testing and then systematically scans the target space, applying the test to all windows of the data up to a given fixed size in time and space [14]. This method has the benefit of being intuitive, but has been criticised for being less efficient than some control chart methods [15]. However, control chart methods such as the MEWMA (Multivariate Exponentially Weighted Moving Average) control chart method proposed by Joner and Woodall *et al*[16], usually do not account for underlying changes such as seasonal effects. Accounting for such underlying effects is crucial when monitoring infectious diseases such as influenza as presentations vary significantly across seasons. Furthermore, while these methods find change points in sets of multiple time series, they do not identify the responsible component series. For example, directional MEWMA can find that the counts for a disease group are increasing, but it cannot indicate that it is mostly caused by say, males under the age of 10.

There are also some multivariate non-parametric approaches, such as Wong et al’s WSARE [17], which compares all possible groups defined by rules of a fixed length with their historic values. This technique becomes very computationally demanding as you increase the rule length. However, it does demonstrate that methods from the machine-learning and data-mining literatures can be exploited in this situation for their ability to find patterns in high dimensional data sets.

The technique explored in this paper, Surveillance Trees, combines aspects from both the machine-learning and control chart literatures. It is inspired by the tree algorithms that are frequently used in machine-learning areas for their ability to seek out patterns in high dimensions and incorporates the benefits of control charts for temporal monitoring by using an EWMA (Exponentially Weighted Moving Average) smoothing. It was originally applied to the problem of monitoring numbers of vehicle crashes [18]. In this paper we explore the particulars of the method and the adjustments required for its application to the problem of ED surveillance.

## Methods

### Setting

The data available from the EDs in this study were in the form of de-identified unit records for each ED presentation from 2006 to 2009 to any of 18 hospitals across Queensland. Approval to use these data was given by the Queensland Health Human Research Ethics Committee (QHREC). Each record was described by variables in three categories: temporal, demographic and presentation type. The variables available in these categories are presented in Table 1.

**Table 1:** Data description

**Information type**	**Variable**	**Variable type**	**Details**
Temporal information	Arrival date and Time	Temporal	To the nearest minute. From 2006 to present day
Presentation information	Facility	Categorical	One of 18 Hospitals spread over QLD (including a children’s hospital)
	Triage category	Categorical (Ordered)	Rating of urgency on presentation: 1,2,3,4,5 (1 requires resuscitation down to 5 being non-urgent)
	Departure Status	Categorical	Discharged, Admitted, Did Not Wait, Transferred, Died in ED, Left Against Advice, Dead On Arrival
	ICD-10 Code	Categorical	A coding of diseases, signs and symptoms, abnormal findings, complaints, social circumstances and external causes of injury or diseases; 5000 unique codes present in data set [19]
Demographic Information	Age	Continuous	Age in years
	Sex	Categorical	

For surveillance we are interested in the number of patients presenting in different groups and how those counts change over time. We essentially transform this unit record data into a large, high dimensional contingency table for each time step. In this table, each *cell* is the smallest possible multidimensional subgroup and has an associated count, that is the number of presentations with a particular disease group, for a particular age, gender, triage category etc. The table is referred to as the *target space* and its rows are the *surveillance variables*. Each cell is considered over time and its collective observations are referred to as a *series*.

The goal was to monitor the behaviour of the cells of the target space as new cell counts were added to each series and to detect, as soon as possible, when counts increased significantly from the expected for any cluster of cells. This approach has the advantage of detecting any clustering of disease instances. It also provides information about the nature of the hotspot by identifying which cells are affected.

As the purpose of this surveillance system is to aid in the management of Emergency Department resources during the winter bed crisis, we limited the analysis to the monitoring of presentations with ICD-10 codes [19] which: 

• have the potential to negatively affect the operation of a hospital during the winter bed crisis (e.g. due to their infectious nature or the sheer volume of cases)

• provide an opportunity for intervention

• have a behaviour which is difficult to predict

or codes that indirectly point to diseases that have the above properties.

After both discussion with clinicians and observation of the seasonal behaviour in data from 2006 to 2008, three ICD-10 code groups were chosen for monitoring. The first was *Flu* related presentations, referred to as the Flu group. The second was *Respiratory* (non-Flu) related presentations, the Respiratory group. The last were presentations listed as *Factors influencing health status and contact with health services* that in exploratory analysis appeared to be particularly prevalent in the winter crisis period. This last group will be referred to as the Factors group. Consultation with domain experts revealed that the *Factors* group were generalist codes that were often used in peak Flu season. For example, code *Z02.7* is defined as “Examination and encounter for administrative purposes: Issue of medical certificate” which increases in prevalence when schools and workplaces require evidence of either fitness or incapacity for attendance or non-attendance respectively. For the remainder of the paper the term ‘Disease Groups’ refers to these three groups of ICD-10 codes.

**Figure 1:**
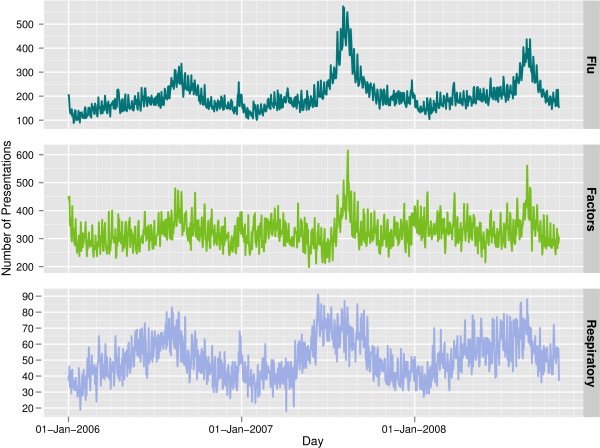
**Daily counts of ED presentations.** For each of the three disease groups being monitored, this figure gives the daily number of presentations across all hospitals. Each series is plotted for the three year period between 2006 and 2008. The seasonal increase in the number of presentations for each group is visible each winter (June-September).

The weekly presentation count across all hospitals for each of the three groups is presented in Figure 1. Table 2 gives the ICD-10 codes that are grouped together to form each Disease Group.

**Table 2:** Disease groups: ICD-10 code groupings

**Disease group**	**Subgroup of ICD-10 codes**	**Description**
	J00-J06	Acute upper respiratory infections
	J09-J18	Influenza and pneumonia
*Flu*: Influenza-associated diseases	J20-J22	Other acute lower respiratory infections
	A00-A09	Intestinal infectious diseases
	B25-B34	Other viral diseases
	J30-J39	Other diseases of upper respiratory tract
	J40-J47	Chronic lower respiratory diseases
	J60-J70	Lung diseases due to external agents
*Respiratory*: Diseases of the respiratory system	J80-J84	Other respiratory diseases principally affecting the interstitium
	J85-J86	Suppurative and necrotic conditions of lower respiratory tract
	J90-J94	Other diseases of pleura
	J95-J99	Other diseases of the respiratory system
	Z00-Z13	Persons encountering health services for examination and investigation
	Z20-Z29	Persons with potential health hazards related to communicable diseases
	Z30-Z39	Persons encountering health services in circumstances related to reproduction
*Factors*: Factors influencing health status and contact with health services	Z40-Z54	Persons encountering health services for specific procedures and health care
	Z55-Z65	Persons with potential health hazards related to socioeconomic and psychosocial circumstances
	Z70-Z76	Persons encountering health services in other circumstances
	Z80-Z99	Persons with potential health hazards related to family and personal history and certain conditions influencing health status

As observed by Chandola et.al. in their survey of outlier detection methodologies [20], most techniques can be reduced to two phases: Phase 1 Determining the probabilistic/predictive model from which the data are generated Phase 2 Testing if instances are consistent with that model or not.

These phases are applicable to the method presented in this paper. So we divide the following sections accordingly, with one section for each phase. Training data, from the period 2006 to 2008, are reserved for both the development of the predictive model, Phase 1, and for training the parameters for the EWMA Surveillance Trees, Phase 2.

### Phase 1: developing a predictive model for patient counts

Before we could apply any testing procedure for unusual behaviour we had first to develop a model for the expected counts of presentations for all series. For example, we needed to be able to forecast the expected number of patients on a given day, for patients of a particular age, at a particular hospital, with a particular disease, etc. For surveillance we want the model to characterise the behaviour of the system when in-control, that is when the behaviour is predictable, and be able to forecast one day ahead with measurable accuracy.

In trying to characterise the behaviour of such a complex system, we felt it important to incorporate the domain knowledge of known behaviours. After discussion with the Director of Patient Flow at Gold Coast Hospital, it was considered that the domain understanding is at two levels. At one level are explanatory variables that have been identified as being useful in predicting the total volume of patient presentations to EDs. Discussion with this expert as well as consideration of previous efforts in inferential modelling [21-23], suggested the inclusion of the following explanatory variables: annual seasonal effects, day of the week contributions, public and school holiday influences, and transitional effects. At the second level, domain practitioners know that there are strong interactions between demographic explanatory variables, such as age, with presentation variables, such as triage category.

As well as incorporating this domain knowledge, we addressed several other challenges: 

• including predictor variables of different types (nominal, ordered categorical and continuous);

• managing the sparsity of the data when we consider counts at such a detailed level of classification;

• modelling the mean of the system and capturing the variation in order to correctly establish unusual cases in the testing phase; and

• adressing the computational challenges posed by the scale of the problem (e.g. even holding the counts in memory for this large target space across many time points is constrained by current memory resources).

In trying to address all of these challenges, we employed a ‘divide and conquer’ approach. Since the domain knowledge of the process of arrivals was at two levels we divided the modelling problem similarly. Rather than one large table to be modelled over time we considered each disease group separately. Then we considered two levels within each group: 

1. Level one involved the total number of presentations for each Disease Group to be modelled over time with forecasts updated each day using a moving window of time; and

2. Level two involved the counts table aggregated over presentation and patient characteristics for the data from 2006 to 2008 that could be used to identify the proportion of daily counts coming from each cell.

To get the expected value for a cell, we used the predicted number of presentations for the whole disease group from the model that used the total number of daily presentations for each Disease Group as the response variable. Then we modelled the proportion of these counts that were expected to be in specific cells, where the response variable was the empirical proportions computed for total counts over the full period 2006 to 2008. The latter model was used to predict the probability that a randomly selected person within a disease group belongs to a partcular cell, e.g, female aged 20, with triage category 1, etc.

#### Step 1: developing a time-dependent model for total counts

For each disease group *i*, we firstly develop a transitional regression model for total counts over time. So let *Y*_*i*_(*t*) be the total volume of patients to that group on day *t*, whose expected value **E**[*Y*_*i*_(*t*)] we model as a function of time using a transitional model with a distribution that is either log-linear poisson or negative binomial:

(1) $$\;\log\left(E[Y_{i}(t)]\right)=\alpha_{0}+\overset{n}{\underset{j=1}{\sum }}\beta_{j}f_{j}(t)+\overset{m}{\underset{k=1}{\sum }}\gamma_{k}\log\left(Y_{i}(t-k)+1\right)$$

Here *f*_*j*_(*t*) are functions of time including seasonal harmonics, or indicators for day of the week, public holidays or school holidays and *Y*_*i*_(*t* − *k*) are lagged, observed counts going back *m* days. The independent variables used for each model are given in Table 3.

**Table 3:** Independent variables of the models for disease group total counts

**Variable**	**Type**	**Description**	**Selected in Flu group model**	**Selected in respiratory group model**	**Selected in factors group model**
*Day*	Continuous	Number of days since beginning of training period in 2006	Yes	Yes	Yes
*Weekday*	Categorical	The day of the week (reference category ‘Monday’)	Yes	Yes	Yes
*sin.day, cos.day*	Continuous	Yearly seasonal harmonics $\text{sin}\;\left(\frac{2\mathrm{\pi Day}}{365}\right)$ and $$\text{cos}\;\left(\frac{2\mathrm{\pi Day}}{365}\right)$$	Yes	Yes	Yes
*log1p.lagn*	Continuous	log of the count for the *n*th day before, *n* = 1,2,…,7, plus 1	Yes	Yes	Yes
*is.public.hol*	Binary	An indicator for whether or not it is a QLD State public holiday	Yes	Yes	No
*is.school.hol*	Binary	An indicator for whether or not it is a QLD State school holiday	Yes	No	No
*l2.mod*	Categorical	The subgroup of ICD-10 codes	Yes	Yes	Yes
l2.mod*Day	Interaction	Interaction between the level 2 disease group and *Day*	Yes	Yes	No
l2.mod*Weekday	Interaction	Interaction between the level 2 disease group and *Weekday*	Yes	Yes	Yes
l2.mod*sin.day	Interaction	Interaction between the level 2 disease group and *sin.day*	Yes	Yes	Yes
l2.mod*cos.day	Interaction	Interaction between the level 2 disease group and *cos.day*	Yes	Yes	Yes
l2.mod*log1p.lag1	Interaction	Interaction between the level 2 disease group and *log1p.lag1*	Yes	Yes	Yes
Weekday* is.school.hol	Interaction	Interaction between the *Weekday* and whether or not it is a school holiday	Yes	No	No
Day*Weekday	Interaction	Interaction between *Day* and *Weekday*	No	Yes	No
Day*sin.day	Interaction	Interaction between *Day* and *sin.day*	No	Yes	No
Day*cos.day	Interaction	Interaction between *Day* and *cos.day*	No	Yes	No
Day*is.public.holiday	Interaction	Interaction between *Day* and *is.public.holiday*	No	Yes	No
Day*is.school.holiday	Interaction	Interaction between *Day* and *is.school.holiday*	No	No	No

The total counts of presentations for each of the disease groups were modelled with these independent variables. The Respiratory group was modelled assuming a Poisson distribution with the Flu and Factors groups were each modelled assuming a Negative Binomial Distribution.

This high-level modelling allows for incorporation of domain knowledge about the timing of presentations and has few computational demands.

#### Step 2: predicting expected proportions to cells

We now need a way to allocate these count totals to all the cells of the target space. In this project we assumed that this allocation remains constant over time and is independent of the total number of presentations.

To model the allocation of counts to cells we use a Poisson Regression Tree approach. We sum the data over time for each cell (each combination of Age, Sex, Triage Category, Facility and Departure Status in the training period from 2006 to 2008) and train a regression tree on these aggregated counts. Let *X* be the set of all cells to be modelled. The resulting tree gives for each cell, **x** = (*x*_1_,*x*_2_,…) ∈ *X*, an expected count, *ν*(**x**), for the whole training period. These estimates are then used as simple proportions, independent of total volume:

(2) $$p(x)=\frac{\nu (x)}{\underset{\xi \in X}{\sum }\nu (\xi )}$$

While the assumption that this allocation remains constant over both time and total volume is unlikely to hold true for most disease groups, little is known about any systematic changes in this process. The advantages of using this regression tree approach are that: 

• by aggregating the data over time we achieve a computationally significant dimension reduction;

• variables of different types are easily included;

• regions of very low or zero frequency are grouped together and are given low (but non-zero) expected values; and

• interactions are naturally included. While these interactions are empirically determined, at the model evaluation stage we can check that the interactions identified by domain experts are captured.

#### Step 3: assigning expected counts to cells

Lastly, for a given cell **x**, disease group *i* and time *t*, the expected number of presentations *μ* is then the product of equations 1 and 2, that is, the product of the expected total count and the proportion to that cell respectively:

(3) $$\mu_{i}(x,t)=E\left[Y_{i}(t)\right]\times p_{i}(x)$$

It is this combination of models that allows us to bypass the computational issues associated with such high dimensional problems. Simultaneously, it allows for the inclusion of domain knowledge.

### Phase 2: testing for unusually high counts using EWMA surveillance trees

We applied the method discussed above to determine the expected means for all possible subgroups of the target space. We now present the method used to detect and diagnose unusually high ED presentation as new ED presentations arrive daily. Usual/expected behaviour in presentations is defined relative to this model’s day ahead forecasts of cell presentation counts.

The Surveillance Tree methodology is a multivariate outlier detection method developed in Sparks and Okugami [18] to monitor numbers of vehicle crashes. At a given time point, the Surveillance Tree method again consists of three major steps to test whether the observed data fit the model of expected counts: 

• applying the EWMA (Exponentially Weighted Moving Average) based temporal smoothing of observed and expected counts;

• growing a Surveillance Tree on departures from expected value in the smoothed counts using a binary recursive partitioning approach;and

• pruning the Surveillance Tree to reveal signals and control the false alarm rate.

The recursive partitioning process is used to decide on the appropriate level of aggregation to best detect the outbreak. It avoids aggregating over sub-dimensions where no outbreak is occurring. Thus it is more efficient than aggregating over the whole multivariate space. Once the best level of aggregation is selected, it remains to test whether the aggregation is significantly unusual to flag as an outbreak.

#### Step 4: EWMA smoothing

Let *y*_*t*_ be the number of presentations on day *t* to a cell **x**. We are given *y*_*t*_, an observed number of presentations, and using a moving window of data up to time *t* − 1 we estimate (forecast) the mean *μ*_*t*_ = *E*(*y*_*t*_) and the variance $\sigma_{t}^{2}=\text{Var}(y_{t})$.

In order to accumulate the temporal memory needed to detect small changes that persist over time, the Surveillance Trees are built based on an EWMA of the observed counts. Let ${ŷ}_{t}$ be the smoothed EWMA of *y*_*t*_

(4) $$\;{ŷ}_{t}=\alpha y_{t}+(1-\alpha ){ŷ}_{t-1}\;\text{for}\;t=1,2,\ldots \text{and where}\;{ŷ}_{0}=y_{0}$$

where *α* is a suitable constant 0 < *α* < 1 that determines how much memory to retain in the average and is usually chosen using training data; in this paper we selected *α* = 0.1. After applying this smoothing to the observed counts we must now consider its effects on the respective mean and variance, so we consider

(5) $$\begin{array}{c} \;{\hat{\mu }}_{t}=\alpha \mu_{t}+(1-\alpha ){\hat{\mu }}_{t-1}\;\text{for}\;t=1,2,\ldots \text{and where}\;{\hat{\mu }}_{0}=\mu_{0} \end{array}$$

(6) $$\begin{array}{c} \;{\hat{\sigma }}_{t}^{2}=\alpha^{2}\sigma_{t}^{2}+{\left(1-\alpha \right)}^{2}{\hat{\sigma }}_{t-1}^{2}\;\;\text{for}\;t=1,2,\ldots \text{and where}\;{\hat{\sigma }}_{0}^{2}=\sigma_{0}^{2} \end{array}$$

In order to begin the testing phase, we need a measure of how far the smoothed counts depart from the expected for any particular level of aggregation. The response variable, *z*_*t*_, considered in this project is the usual z-score standardisation to a statistic with mean zero and variance one:

(7) $$z_{t}=\frac{{ŷ}_{t}-{\hat{\mu }}_{t}}{{\hat{\sigma }}_{t}}$$

For any cell or aggregation of cells, the measure of departure from expected is calculated using this formula - combining the sum of smoothed counts in the cells, the sum of smoothed predictions for those cells, and the variance of the smoothed counts for those cells.

#### Step 5: growing the surveillance tree

The response variable *z*_*t*_ is then used to grow a Surveillance Tree at each time point. The Tree is grown using a binary recursive partitioning approach whose goal is to identify regions in the target space with unusually high departures from expected counts.

The process begins with the whole target space and the focus for each partition is to find a region with (in some sense) an unusually high value of *z*_*t*_. At each stage of the tree growing process, we consider a parent region of the target space. For this region we calculate the value of the test statistic for all sub-regions that can be generated by taking binary partitions along any surveillance variable. The partition which maximises the test statistic is chosen and the parent region is split on that variable into two offspring. Of these two offspring, one is that with the maximising test statistic and the other is simply the remainder of the parent region. The process is then repeated considering each of the two offspring as parents. Each generation of offspring is grown in the same way and gives rise to a representation of the target space by means of a tree data structure referred to as a *Surveillance Tree*. 

**Figure 2:**
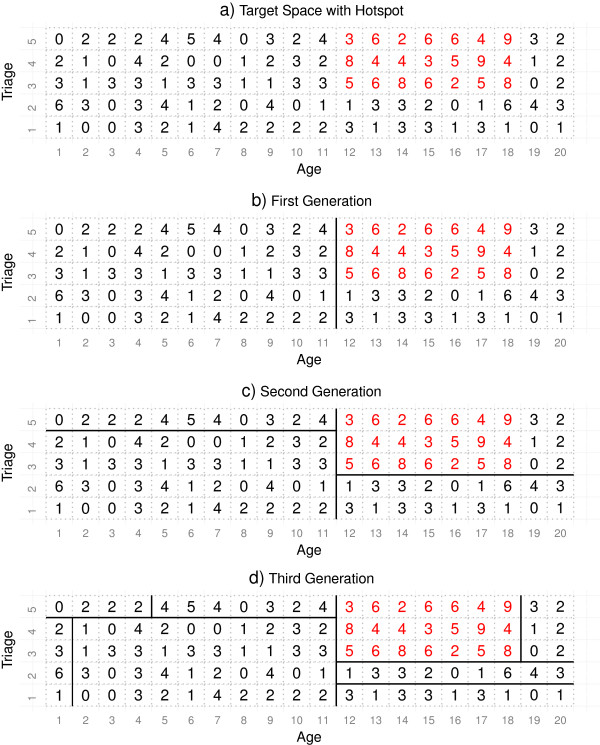
**An example of recursive partitioning for 2 variables.** An example of the tree growing procedure over two variables. In this case each cell has an expected poisson count with mean 2. The target space is shown in panel **a)** with hotspot illustrated in red. The first generation partition is in panel **b)**. Each of the two second generation partitions is in panel **c)**. And each of the four third generation partitions are in panel **d)**.

Consider an example with only two surveillance variables: Triage Category which has possible values of 1,2,…,5 and Age which in this example has possible values of 1,2,…,20. For simplicity, assume that each cell in this target space (each age and triage combination) is expected to be Poisson distributed with a mean of 2. Counts that might be observed are given in panel a) of Figure 2. In this case all the cells labelled in black are indeed generated from a Poisson distribution with mean 2. However, we have added a hotspot for the subspace of ages 12 to 18 and triage categories 3 to 5. These cells are illustrated in red text and have been generated from a Poisson distribution with mean 6. Again for simplicity in this example we ignore the EWMA smoothing described above.

**Figure 3:**
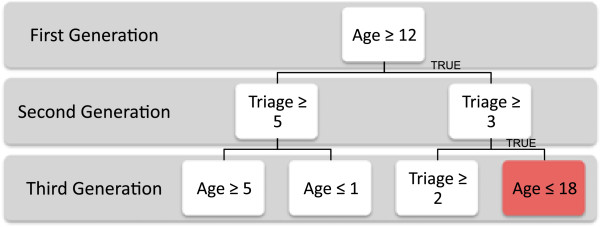
**An example of a full Surveillance Tree before pruning.** This is the tree representation of the partitioning from Figure 2. The node which describes the region of the hotspot is shaded in red.

For the example in Figure 2, the recursive partitioning starts by searching for the best partition of Age and Triage Category which results in a region that maximises the departure of the counts from the region’s expected value, that is, maximises *z*_*t*_. Note that since the cell expected value in Figure 2 is 2, then $u_{t}=\sigma_{t}^{2}=2$. In this example the best partition on the Age variable is *Age* ≥ 12 and that on the Triage variable is *Triage* ≥ 3. Of these two possible partitions, it is the split on Age that maximises *z*_*t*_. Panel b) of Figure 2 shows this choice of split which generates the first generation of offspring. Each of the two regions now shown in panel b) become parents. Each grows two offspring by finding the partition that is best in each case. For the parent region with *Age* ≥ 12, it is clear that the best partition on either Age or Triage is *Triage* ≥ 3. For the region with *Age* < 12, the best partition is less obvious - it turns out to be *Triage* ≥ 5. Panel c) in Figure 2 shows these two new splits and the next generation of offspring. One final round of partitioning is given in panel d), which gives the generation that completely specifies the simulated outbreak. The z-score for the red region is $z_{t}=10.96=(3+6+\ldots +5+8-3\times 7\times 2)/\sqrt{3\times 7\times 2}$ which is higher than any other partitioned region in Figure 3.

The tree representation of the example given in Figure 2, is given in Figure 3. The node shaded in red describes the region of the hotspot. Once partitioning has stopped, then recursive pruning of the terminal nodes commences. The pruning process is outlined in the next section. If after pruning this red node remained then this would signal this subspace as a possible hotspot for further investigation.

In this example we used only ordered variables, but we note that for unordered categorical variables, Sparks and Okugami [18] provide a method for finding partitions without searching all possible binary splits. The method involves ranking the categories of the variable by their *z*_*t*_ and then treating the variable as if it were ordered.

As used in the example above, the naive test statistic for each partition is simply *z*_*t*_ itself. However, we have variables of different types and sizes. For example the variable ‘Gender’ has only one possible partition whereas the variable ‘Age’ has over 100. To make the variable selection process equally likely for each variable, we use the same approach as in Sparks and Okugami [18]. We generate parametric bootstrap samples from the model of in-control behaviour over time and grow Surveillance Trees on these samples. The result is data on the range of values for maximising *z*_*t*_ scores for in-control situations.

Those data are used to model, for each variable, the location and spread of in-control *z*_*t*_ scores conditional on variables such as the amount of searching, ${\hat{\mu }}_{t}$ and *z*_*t*_ in the parent, and ${\hat{\mu }}_{t}$ in the node itself. Thus, if *μ*^∗^ and (*σ*^∗^)^2^ are the respective conditional estimates of mean and variance, then the final test statistic used to choose partitions is

(8) $$z_{t}^{*}=\frac{z_{t}-\mu^{*}}{\sigma^{*}}$$

The growing of the Surveillance Tree continues until stopping criteria are met. (In this paper growing was stopped either when the node-smoothed count was less than 4 or a maximum tree depth of 10 levels of partitioning was reached. These thresholds were chosen because it was felt that an outbreak signalled beyond either these criteria would be of little use to practitioners from an operational perspective.) Once partitioning has been completed, then recursive pruning of the terminal nodes commences.

#### Step 6: pruning the surveillance tree

The aim of pruning is to trim away all insignificant nodes. If all nodes in the tree are pruned away for a particular time point then nothing is signalled. However, if nodes remain after pruning is completed, then an alarm is given. The location of the hotspot within the population is diagnosed by the set of partitioning rules that define the remaining terminal nodes.

Again, the pruning process is given in more detail in Sparks and Okugami [18] but perhaps the most important aspect of the pruning strategy is the one designed to control the false alarm rate. Nodes are pruned recursively starting with the last offspring in the tree. Nodes are pruned, that is dropped, if their *z*-score fails to exceed an upper threshold value *τ* which is a function of the properties of the node. So node *n* with *z*-score *z* is dropped if 

1. *z* < *τ*(*n*) or

2. *pz* > *τ*(*pn*) and *z* < *pz* where *pn* is the parent node of *n* and *pz* its corresponding *z*-score.

This threshold *τ*(*node*) is used to control the false alarm rate and adjusts for the properties of the node such as the mean and depth in the tree. In addition, it differs for each variable because some variables are continuous (but on different scales) while others are categorical (but with different number of categories).

In order to determine *τ* such that the pruning of nodes is conditionally independent of the properties of the nodes themselves (node mean *μ* and node depth *ν*), we use bootstrapped, in-control samples from the model for training. Using these samples, we run simulations of the EWMA Surveillance Tree partitioning. We then use the data generated from each partition in each of these simulations (*z*-score, partitioning variable, expected count and depth of the winning partitions) to train the coefficients of a threshold model that gives the desired false alarm rate.

In this paper, we used the following model formulation for *τ*, where *μ*_*n*_ is the mean for node *n* and *ν*_*n*_ is the depth of node *n* in the tree:

$$\begin{array}{ll} \;\tau (n) & =a_{0}+a_{1}\mu_{n}+a_{2}\nu_{n}+a_{3}\frac{1}{\mu_{n}}+a_{4}\nu_{n}^{2}+a_{5}\frac{1}{\nu_{n}}\\ & \;\;+a_{6}\mu_{n}\nu_{n}+a_{7}\mu_{n}\frac{1}{\nu_{n}} \end{array}$$

Different coefficients *a*_*i*_ were estimated for each partitioning variable to ensure that each variable is equally likely to deliver a false alarm signal thus not biasing the threshold to flag specific clusters over others. The terms of the model were chosen by observing properties of the simulated *z*-scores with respect to their corresponding partitioning variable, mean and depth in the tree. Quantile regression was used on the simulation data to create starting values for the coefficients for *τ* for each partitioning variable. These models were then checked using simulation, and then their intercepts raised iteratively by a fixed amount until the pruning resulted in the goal rate of approximately 3 false alarms per year and each variable approximately equally likely to signal. The coefficients for all models are given in Table 4. The result of applying these threshold models over 1000 in-control bootstrapped samples (with no outlier removal) was an average time-to-signal of 134.65.

**Table 4:** **Coefficients of the models of the pruning threshold *τ * across variables**

	**Age**	**Sex**	**Triage category**	**Departure status**	**Facility**	**Disease group**	**Disease subgroup**
(Intercept)	3.7598	3.6926	3.9562	3.9712	3.3100	4.1558	3.5891
*μ*	0.0000	-0.0022	-0.0012	-0.0007	0.0017	-0.0012	-0.0015
depth	-0.1481	-0.1772	-0.2327	-0.2209	-0.0675	-0.2605	-0.1022
1/ *μ*	0.6342	1.2781	0.6233	0.5121	0.8856	0.9699	0.0327
depth^2^	0.0060	0.0076	0.0121	0.0108	0.0015	0.0121	0.0038
1/depth	-0.9182	-0.8120	-1.0811	-1.0864	-0.4693	-1.2520	-1.3850
μ*depth	-0.0006	-0.0001	-0.0003	-0.0003	-0.0008	-0.0002	-0.0006
μ*(1/depth)	0.0004	0.0021	0.0014	0.0010	-0.0009	0.0013	0.0043

Given here are the results of the quantile regression used to choose a threshold for each variable such that each variable is equally likely to signal; signalling is independent of node expected value *μ* and depth in the tree; and the overall false alarm rate is approximately 3 times per year.

#### Applying the test prospectively

Once the parameters of the Surveillance Trees have been determined from the training data, then given an incoming stream of new presentations for testing, we proceed as follows. For each day *t*, we calculate the counts *y*_*t*_ of presentations for each cell and apply an EWMA-smoothing to them to give ${ŷ}_{t}$ (see Equation 4) for each cell. We re-estimate the temporal model using the 3 year window of data up to *t* − 1. Then we provide a day-ahead forecast of expected counts and variances to disease groups for day *t*. These estimates are then allocated proportionally to all cells to give the expected cell count and variance to cells, i.e. *μ*_*t*_ and *σ*_*t*_ respectively. These are adjusted for the smoothing according to Equations 5 and 6 to give ${\hat{\mu }}_{t}$ and ${\hat{\sigma }}_{t}$.

A Surveillance Tree is then grown using the standardised *z*-score calculated in equation 8 to choose partitions. Once grown the tree is then pruned according to the rules above and the threshold *τ*(*node*). If all nodes are pruned away then no signal is given. If anything remains, a hotspot is signalled and the branches of the tree left un-pruned describe its location.

### Evaluation of the methodology by simulation

In order to ascertain the sensitivity of the methodology, we applied the system to various simulated, artificial increases in the number of disease presentations or ‘hotspots’. This simulation approach allowed for assessment of the effectiveness of the methodology (how often it successfully detects a hotspot), its timeliness (how long it takes after a hotspot is introduced to be detected) and its diagnostic capability (how accurately it describes the population affected by the hotspot). Furthermore, application of the methodology to simulated data allows us to compare its effectiveness and timeliness with an example of a currently used tool: a univariate EWMA control chart with adjustments for expected values [13,21].

To create the simulation data sets, 1000 bootstrapped, in-control time series for 2009 were created using the models developed on the 2006 to 2008 data. Since outbreaks might occur at any time of year, the artificial outbreak being tested was added to each sample at a randomly selected start date in 2009. This random selection also allowed us to judge whether time-of-year of the outbreak has an effect on sensitivity of the methodology. The influences of starting the outbreak at different times of the year, and different sizes of outbreaks are investigated in section “The effect of hotspot strength, duration and timing” later. Since the clustering nature of outbreaks can vary from outbreak to outbreak, hotspots that cluster in different subspaces are investigated in the section “The effect of hotspot clustering”.

Each simulated run produces a different in-control sample (*in-control bootstrap sample*) and similarly the artificial hotspot data (*out-of-control bootstrapped sample*) differs for each simulation run even though it is generated with the same parabolic mean counts (see section “Simulated Hotspots” below). The Surveillance Tree methodology was then run on each out-of-control bootstrapped sample using a burn in period of 20 days before the start time of the artificial hotspot to allow for the EWMA smoothing process to reach a steady state.

#### Simulated hotspots

Once the subspace that the hotspot affects was established, a negative binomial distributed random count was simulated each day and added to the respective subgroup. The mean was changed to emulate an infectious outbreak by modifying it according to a parabola which is itself described by parameters ‘peak height’ and ‘peak day’. So a hotspot with peak day 7 and peak height 20 has a distribution whose mean starts at 0 on day 0, increases at a quadratic rate to a value of 20 on day 7 and similarly decreases until it is 0 again on day 15. All hotspots used in this paper assumed a dispersion parameter of 10.

The choice of subspace was made to capture plausible scenarios in an Emergency Department context, but also to test a number of different aspects of the surveillance problem. See ‘Results and Discussion’ below for descriptions of the hotspots tested and for the trait of the method being tested.

#### Evaluation measures

The first of the evaluation measures addessed the measure effectiveness and timeliness of the methodology. Effectiveness was measured by looking at the percentage of simulations where a hotspot was successfully detected over the period that the hotspot is being applied. In order to measure timeliness, that is how quickly a hotspot is detected, we used the time-to-signal as suggested in [15]. This is the number of days from the known introduction of the hotspot to the time when it is signalled. The second evaluation measure examined the influence hotspot strength, duration and timing have on effectiveness and timeliness. The final measure examined the diagnostic properties of the Surveillance tree methodlogy.

## Results and discussion

### Surveillance trees compared to univariate control chart in terms of effectiveness and timeliness

For each hotspot location and scenario, the increase in subgroup counts were added to each of the 1000 simulated in-control samples. These then underwent the evaluation process for both the Surveillance Tree test and a univariate control chart for comparison. The univariate control chart used in this paper was an EWMA control chart of Flu presentations, referred to subsequently as the univariate control chart, which monitors total flu counts departures from their expected value, where the expected values and variances are calculated exactly as for the Surveillance Tree. The EWMA smoothing parameter was also set at the same value of 0.1. The univariate control chart was trained to have approximately the same false alarm rate as the Surveillance Trees (135.32 and 134.65 respectively, achieved over 1000 in-control bootstrapped samples). The training of the Surveillance Tree is described in Step 6 above. The univariate control chart was trained by setting its threshold parameter such that it achieved approximately the same false alarm rate over the 1000 simulated samples (in this case the parameter that multiplies the standard deviation to establish the upper control limit was 2.38). Both were achieved using the full training sets with no outlier removal since in both cases, the EWMA smoothing is expected to minimise the effects of one-off outliers in the time series.

Since the Surveillance Trees methodology is a multivariate chart, it is unknown what the equivalent time-to-signal should be in order to create a comparable univariate chart. We decided to use the same in-control time-to-signal as the multivariate chart but recognise that this criterion offers an unfair comparison (in favour of the univariate chart). The presumable advantage to the univariate chart is that both methods are trying to detect a signal of the same strength but the univariate control chart assumes the hotspot is in the Flu group counts (top panel, Figure 1) whereas the Surveillance Tree method does not make this assumption and tries to detect it out of all counts, as illustrated in Figure 1. Furthermore, the false alarm rate for the univariate control chart is for the monitoring of only one series, whereas that of the Surveillance Tree method is for all subgroups. The univariate charts were expected to perform better for flu related outbreaks for this reason.

In order to demonstrate the capabilities of the methodology in a real world setting, we applied it to real data as though they were coming in online. As test data, we used the ED presentations of 2009.

#### The effect of hotspot clustering

We wanted to firstly compare the sensitivity of the Surveillance Tree method with that of the univariate control chart method for hotspots across different subspaces and of different sizes. Specifically, we wanted to address two scenarios: 

• The hotspot affects the whole population being monitored by the univariate control chart, i.e. there is no clustering of the higher counts in a subspace. In other words, what do the Surveillance Trees lose in performance when we are in the optimal situation for the univariate control chart?

• The hotspot affects a subgroup of the population being monitored by the univariate control chart. In other words, what do we gain by using the Surveillance Tree method to search for subgroups?

**Figure 4:**
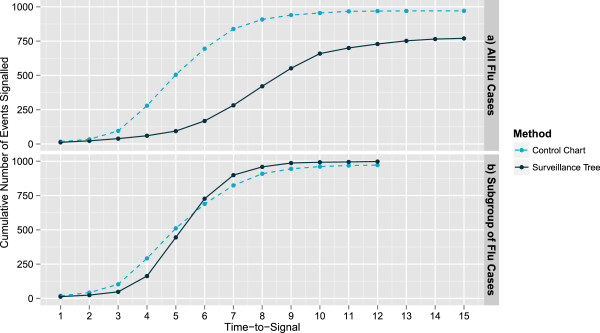
**Cumulative count of hotspots detected for two hotspot types.** This presents the cumulative number of simulations where the hotspot is successfully detected by each method over the known period of the hotspot. Figure 4**a** is for the case where the hotspot affects all Flu presentations (Peak height 40 at day 7). Figure 4**b** is for the case where the hotspot affects only Flu patients aged between 2 and 12 who are admitted as inpatients at the conclusion of their presentation (Peak height 40 at day 7).

We considered two hotspots, one across all Flu presentations and one affecting only Flu patients aged between 2 and 12 who were admitted as inpatients at the conclusion of their presentation. The results of simulating these two hotspots 1000 times and using each method for testing are presented in Figure 4. It is clear that across all flu presentations, (Figure 4a), the univariate control chart outperforms the Surveillance Tree in both the number of times it successfully detects the hotspot and how quickly they are detected. This situation is biased towards the univariate control chart as it is designed appropriately for this level of aggregation and by the fact that the multivariate Surveilance Tree methodology has an overall false alarm rate equivalent to this univariate chart. Table 5 shows that by the peak of the hotspot the univariate control chart has detected over 80% of cases while the Surveillance has detected less than 30%. However, if the hotspot is limited to a subgroup as in Figure 4b, the Surveillance Tree method catches up with respect to both the number of cases detected and the timeliness of detection.

**Table 5:** Simulation results for hotspots across the whole Flu group and a subgroup

**Hotspot**	**Number found by hotspot peak**		**Number found by hotspot end**	
	***Univariate control chart***	**Surveillance tree**	***Univariate control chart***	**Surveillance tree**
a. All flu cases	839	282	971	770
b. Subgroup of flu cases	824	899	972	998

The performance results for each method over the 1000 simulations for two hotspots: one over the whole Flu disease group, the other over the subgroup of Flu cases aged 2-12 and with departure status admitted.

**Figure 5:**
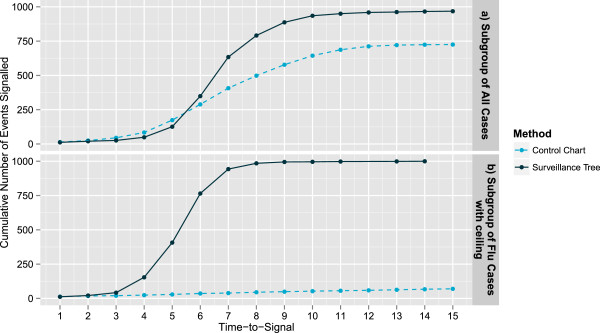
**Cumulative count of hotspots detected for two hotspot types.** This presents the cumulative number of simulations where the hotspot is successfully detected by each method over the known period of the hotspot. Figure 5**a** is for the case where the hotspot affects patients aged between 2 and 12 who are admitted as inpatients at the conclusion of their presentation across all 3 the disease groups (Peak height 40 at day 7). Figure 5**b** is for the case where the hotspot affects only Flu patients aged between 2 and 12 who are admitted as inpatients at the conclusion of their presentation with other Flu patients presenting in relative fewer numbers (Peak height 40 at day 7).

In both scenarios, the control chart performs identically because in both cases the total Flu counts are the same, but in the second case (Figure 5b) the counts cluster. However, the Surveillance Tree method takes advantage of the clustering in Figure 5b and is able to improve its performance by finding the appropriate level of aggregation. We note that in Figure 5b the Surveillance Tree method does not flag the potential outbreak early in its development (days 1 to 5) because it has not gathered enough information to estimate the appropriate level of aggregation. But after a time-to-signal of 6 or more days, it is estimating the appropriate level of aggregation and is able to signal a hotspot more frequently than the univariate control chart.

There are two further scenarios where the Surveillance Tree will also have an advantage. Firstly, where the hotspot is ‘poorly specified‘ and so presents across a broader variety of diagnosis codes. Note that a variety of assigned diagnosis codes across categories is commonplace in syndromic surveillance and is a commonly accepted problem of surveillance methods based on Emergency Department diagnosis codes [24,25]. For example, in this application a presentation might be coded in a number of different ways: ‘Acute upper respiratory infection’ or ‘Persons encountering health services for examination and investigation’ if they are there for a medical certificate for exemption from work/school. If related presentations are spread across codes a univariate surveillance system will miss cases. We illustrate this weakness of univariate monitoring in Figure 5a with a hotspot that again affects patients aged between 2 and 12 who were admitted as inpatients at the conclusion of their presentation, but this time the hotspot occurs across all 3 of the disease groups monitored by the Surveillance Trees.

A second case is where the hotspot affects a subgroup of the population but due to, for example, the limited capacity of the Emergency Departments, the total population does not increase. In this case we suppose there is a hotspot again affecting Flu patients aged between 2 and 12 who are admitted as inpatients at the conclusion of their presentation, but this time we assume that other patients (for example less serious cases) either exist in fewer numbers or just do not present. This effect on a subgroup without changing the total is illustrated in Figure 5b and some results are also given in Table 6.

**Table 6:** Simulation results for hotspots across multiple disease groups and across a subgroup with no aggregate change

**Hotspot**	**Number found by hotspot peak**		**Number found by hotspot end**	
	***Univariate control chart***	**Surveillance tree**	***Univariate control chart***	**Surveillance tree**
a. Subgroup of All Cases	407	634	725	968
b. Subgroup of Flu Cases with ceiling	39	943	70	1000

The performance results for each method over the 1000 simulations for two hotspots: one over the subgroup of cases aged 2-12 and with departure status admitted across all 3 disease groups and the other over the same subgroup in only Flu cases but with a corresponding decrease in other Flu cases.

In both of the above cases, as shown in Figure 5, the univariate control chart is at a disadvantage as the aggregated numbers either do not increase much (as in the first scenario where cases are spread between disease groups) or in the extreme, effectively do not increase at all (as in the second scenario). In both cases the performance of the Surveillance Tree is superior.

#### The effect of hotspot strength, duration and timing

As well as the effect of different hotspot types, we also considered the effect of changing a number of hotspot parameters. The hotspot type used in the following sections was kept fixed: there were increased counts across the three disease groups for all patients that are aged between 2 and 12 and who are admitted as inpatients at the conclusion of their presentation.

**Figure 6:**
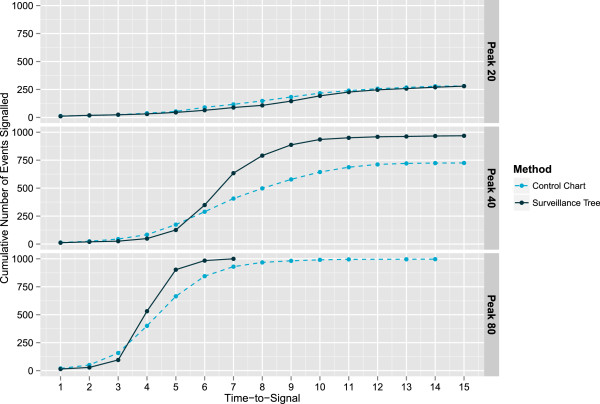
**Cumulative count of hotspots detected for hotspots of different peak heights.** This presents the cumulative number of simulations where the hotspot is successfully detected by each method over the known period of the hotspot. Each panel is for a hotspot of different peak height (20, 40 and 80 on day 7) but of the same type (a hotspot that affects patients aged between 2 and 12 who are admitted as inpatients at the conclusion of their presentation across all 3 the disease groups).

Firstly, the influence of the height of the hotspot peak was explored and Table 7 and Figure 6 show results. For a smaller hotspot (Peak height 20), neither method performs very well, with both detecting fewer than 30% of cases in total. For a mid-sized hotspot (Peak height 40) the Surveillance Tree has detected over 60% of cases by the peak of the hotspot and for a large hotspot (peak height 80), by the peak all cases have been detected. These results again demonstrate that when the information is sufficient to estimate the appropriate level of aggregation, the Surveillance Tree method performs relatively better.

**Table 7:** Simulation results for hotspots with changing peak height

**Hotspot**	**Number found by hotspot peak**		**Number found by hotspot end**	
	***Univariate control chart***	**Surveillance tree**	***Univariate control chart***	**Surveillance tree**
Peak 20	117	89	281	280
Peak 40	407	634	725	968
Peak 80	930	1000	997	1000

The performance results for each method over the 1000 simulations for the same hotspot type but with changing peak height.

**Figure 7:**
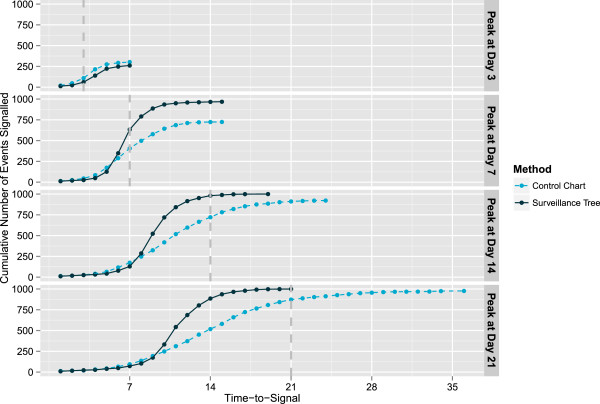
**Cumulative count of hotspots detected for hotspots of different temporal widths.** This presents the cumulative number of simulations where the hotspot is successfully detected by each method over the known period of the hotspot. Each panel is for a hotspot of different duration (Peak height 40 on day 3, 7, 14 and 21 respectively) but of the same type (a hotspot that affects patients aged between 2 and 12 who are admitted as inpatients at the conclusion of their presentation across all 3 the disease groups).

Next are presented the results of changing the duration with hotspot type as above and peak height fixed at 40. The results in Table 8 and Figure 7 show that as the hotspot is lengthened both methods are able to detect higher and higher proportions of the cases by the hotspot peak time. In both cases this detection success is due to the EWMA portion of both algorithms that builds in temporal memory. On the other hand, performance for a short hotspot (peak height achieved at day 3) is poor in both cases. To some extent this detection property can be tuned through the choice of EWMA smoothing parameter *α*. But for the purpose of reacting to an outbreak of disease, hotspots of short duration are of little interest as there isn’t enough time to detect, diagnose and implement a change before the outbreak resolves itself naturally.

**Table 8:** Simulation results for hotspots with changing duration

**Hotspot**	**Number found by hotspot peak**		**Number found by hotspot end**	
	***Univariate control chart***	**Surveillance tree**	***Univariate control chart***	**Surveillance tree**
Peak at Day 3	109	61	303	261
Peak at Day 7	407	634	725	968
Peak at Day 14	721	981	922	1000
Peak at Day 21	873	1000	977	1000

The performance results for each method over the 1000 simulations for the same hotspot type but with changing hotspot duration.

**Figure 8:**
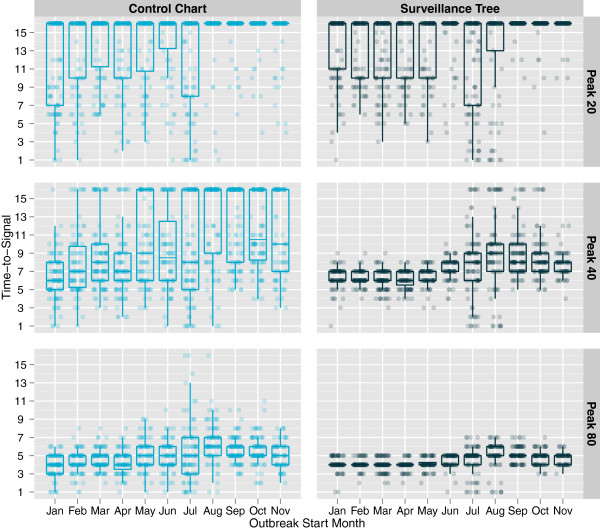
**Distribution of time-to-signal by hotspot start month for hotspots of different peak heights.** This presents boxplots of the time-to-signal for each simulated hotspot grouped by the month in which the hotspot started. Each horizontal panel is for a hotspot of different peak height (20, 40 and 80 on day 7) but of the same type (a hotspot that affects patients aged between 2 and 12 who are admitted as inpatients at the conclusion of their presentation across all 3 the disease groups).

Lastly, the timing of the hotspot relative to normal seasonal peaks and troughs was considered. Figure 8 shows the effect on timeliness of detection for 3 different peak heights and plotted by month in which the hotspot start date occurred. In this plot, in cases where no hotspot was detected, the data point is given a time-to-signal of 16 (one day more than the maximum time allowed for detection). Regardless of peak height, or method, the most variable detection times occurred for simulations where the hotspot started in the months of June, July, August and September. This variability in detection times is not surprising because of the year-to-year variance observed in the onset of the seasonal increase. Determining whether one method is more affected by the timing of the hotspot is difficult because their overall performance is so different. However, the timing effect does not appear particularly worse for any particular method.

### Diagnostic ability

In situations where disease is likely to cluster in unknown subpopulations then the Surveillance Tree method has an advantage in sensitivity over the univariate control chart. However, a further benefit of the Surveillance Tree method is that not only is the hotspot detected, but some information results from the method that can aid in the diagnosis of who is affected. With the simulations described above, because the actual affected subspace is known, we can compare the result of the subspace signalled, say *g*_1_, with the true affected subspace, say *g*_2_. For example, suppose the hotspot is for patients aged between 2 and 12 as above, but the signal is for patients aged 1 to 15. We can compare the actual subspace with the signalled subspace and assess the accuracy of the signal.

A quantitative measure of assessment is to look at a measure of correlation *ρ* between the two populations *g*_1_ and *g*_2_:

(9) $$\rho (\;g_{1},g_{2})=\frac{V(\;g_{1}\cap g_{2})}{\sqrt{V(\;g_{1})V(\;g_{2})}}$$

where *V*(*g*_*i*_) can be thought of as the number of cells included in the subspace *g*_*i*_. If we are trying to estimate the amount of overlap between two subspaces, then *V*(*g*_1_∩*g*_2_) is the number of cells common to both *g*_1_ and *g*_2_. So *ρ* provides a measure of overlap between the two subspaces.

**Figure 9:**
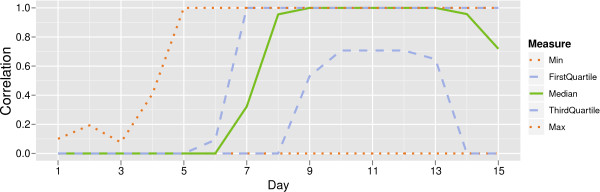
**Five value summary of the distribution of correlation values between signal and true hotspot.** Here we consider the correlation between the true hotspot and the signalled populations. The plot gives 5 statistics summarising the distribution of these correlations values: the minimum, the first quartile, the median, the third quartile and the maximum. Simulations where no hotspots are detected are given a correlation value of 0.

Figure 9 shows the distribution of correlation values changing over the monitoring period for the hotspot with peak height 40 as used above. By the peak height, over 25% of hotspots had been detected with perfect correlation 1.

**Figure 10:**
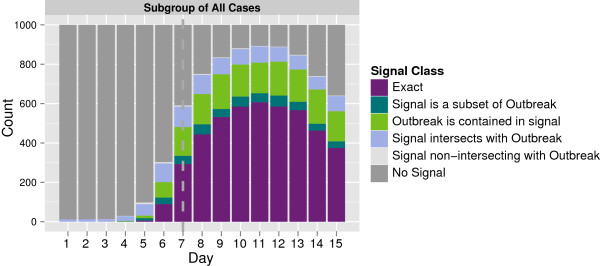
**Count of hotspots detected by types.** This plot illustrates where the signalled population lies in the target space with respect to the true affected subpopulation.

Diagnosis can also be considered more qualitatively by considering where the signalled population lies with respect to the true affected subpopulation: exactly coincides, subset within, superset without, intersecting or non-intersecting. This uncertainty in capturing the truly affected population is illustrated in Figure 10 for the same hotspot, considering the classification of each simulation at each time point in one of those categories. The increase in quality of signals can be seen as well as the fact that for the most part, imperfect matching is due to over- or under-specification rather than a misaligned description of the population. The example in Figure 10 demonstrates that in more than 50% of the cases from day 9 to day 15, the hotspot is identified precisely. From day 7 onwards, the process either completely defines the hotspot or the hotspot is contained within the signalled subspace more than 50% of the time.

Information about the diagnosis of a hotspot is crucial to the next step in the quality improvement cycle and the information provided by the Surveillance Trees provides a useful starting point for this assessment. Once the affected group is confirmed, steps can be taken to cope with the demands of that particular subgroup. Steps might include the establishment of separate Flu clinics for infectious patients or a temporary increase in specialist care such as using paediatric or geriatric resources. In some cases, it might also allow for targeted communication with subsections of the public to ensure ED resources are being used appropriately.

### Illustrative application

**Figure 11:**
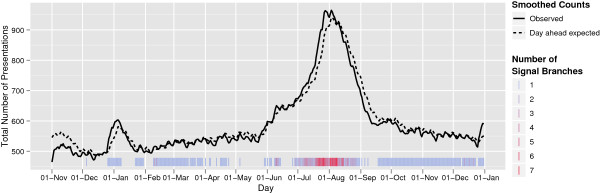
**Timing of Surveillance Tree signals for 2009 test data.** The solid black line in this figure gives the daily, smoothed number of presentations observed overall over 2009. The dashed line gives the smoothed day-ahead forecasts of the expected count. The coloured tick marks along the bottom of the diagram indicate when the Surveillance Tree system has signalled. At one end of the colour spectrum, a blue bar indicates one tree branch was left after pruning, at the red end of the spectrum there where 7 branches left.

When Surveillance Trees are applied to real-world data, there are a number of further considerations to be made. Figure 11 provides a summary of when signals occurred when the Surveillance Trees were applied to the real data of 2009, the year of the H1N1 flu outbreak. This figure shows the daily, smoothed number of presentations observed overall, along with the smoothed day-ahead forecasts of expected count. The coloured bars along the bottom of the diagram indicate when the Surveillance Tree system has signalled. At one end of the colour spectrum, a blue bar indicates one tree branch was left after pruning; at the red end of the spectrum there where 7 branches left. Note that signals occur even when the total number of presentations is below expected, indicating that even when the whole is not unusual some subspace is found to be appearing in greater numbers than expected.

**Figure 12:**
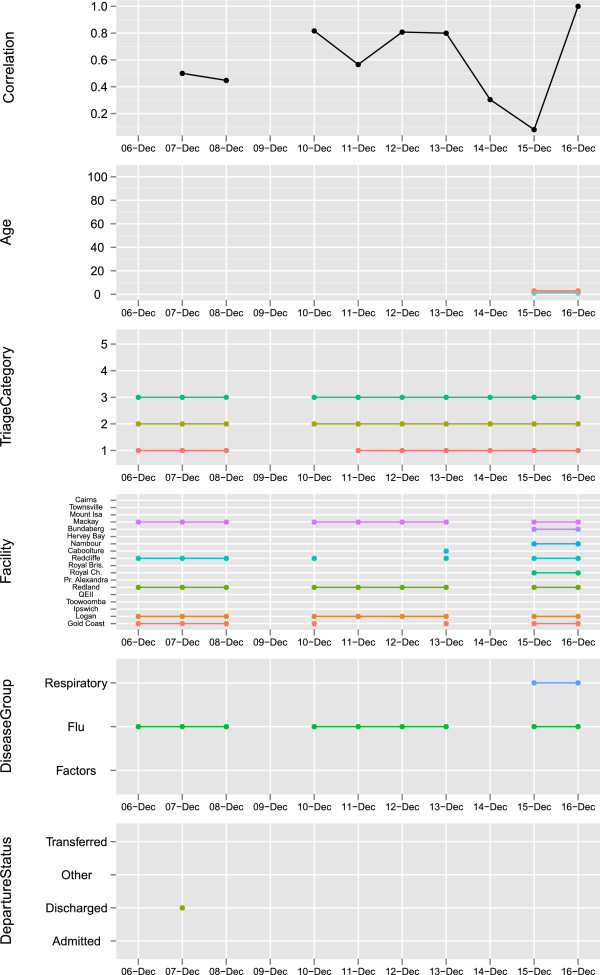
**Signal properties for the period from the 6th to the 12th of December.** An example of how to view the results of the multivariate signals as they progress over time. The figure shows the signals for the period between the 6th and 16th of December 2009. There was a signal everyday in this period except for December 9th. The figure shows for a given day, the correlation (if there was a signal the previous day) and which elements of each of 4 displayed variables were indicated by the signalled hotspot.

This introduces an issue of interpretation. When periods occur with signals day after day we must determine whether these signals are due to the same underlying group being signalled each day, or whether multiple groups are being identified. One method considered is to monitor correlation of the signalled group with that of the previous day, as well as to monitor which variables are being signalled and in what way. Figure 12 presents an example of a way to visualise such monitoring. This figure shows the resulting signals for the period between the 6th and 16th of December. There was a signal every day in this period except for December 9th. The figure shows, for a given day, the correlation as calculated by Equation 9, where *g*_1_ is the current day’s signalled subspace and *g*_2_ is the previous day’s signalled subspace (if there was a signal the previous day).

Figure 12 also provides which elements of each of the 4 displayed variables were indicated by the signalled hotspot. For example, on December 6th there was a signal that indicated a hotspot for the following subspace: the Flu Disease group, Triage Category 1, 2 or 3 and Facility in either Gold Coast Hospital, Logan Hospital, Mackay Base Hospital, Redcliffe Hospital, or Redland Hospital. This group has a smoothed expected count of ${\hat{\mu }}_{t}=33.25$ but instead had ${ŷ}_{t}=49.63$ (where *t* = “2009-12-06”). We can see that while the signal is not exactly the same day by day, with some elements coming in and out, the correlation remains high. For example, the following day, December 7th, there is a signal for essentially the same subspace but with the additional criteria that the presentation ends with patients being discharged. With this additional condition the group has a smoothed expected count of ${\hat{\mu }}_{t}=23.41$ but instead had ${ŷ}_{t}=39.04$ (where *t* = “2009-12-07”). The correlation of signals remains high until around the 14th or 15th of December when there is a drop in correlation and we can see in Figure 12 that there has been a change in the elements being signalled. We could consider therefore that the hotspot responsible for the second week’s display, and hence the disease process may have changed.

The signals for 2009, as given in Figure 11, prompt a number of issues for further, retrospective analysis. Firstly, a group of facilities signalled frequently around public holidays and in the summer months suggesting a spatio-temporal interaction not captured in the model. Secondly, the 31st of May saw the first of a series of school closures in Queensland due to efforts in controlling the spread of the Swine Flu pandemic (Influenza H1N1). Around this time we see an increase in the frequency and complexity of signals that persists through the winter. These signals were complex with many interacting variables but some notable features were that signals tended to be for less serious cases (cases who were discharged at the end of their presentation and/or who presented with triage categories 3, 4 or 5) with age groups signalled frequently between 4 and 50. The fact that the swine flu pandemic disproportionately affected the young is a known feature [8,26], with research suggesting that people over 60 had some acquired resistance from exposure to a previous strain [27]. The findings from the Surveillance Tree analysis would require further retrospective analysis for confirmation in this data set.

## Conclusion

The early detection of changes in presentations to hospital EDs is an important part of any suite of management tools aimed at time and resource efficiency. We have demonstrated that the Surveillance Tree methodology presented in this paper addresses the problems of implementing such a surveillance method for ED surveillance. Traditional univariate approaches such as an EWMA chart will always have the advantage if the population of interest is known in advance. However, since this is frequently not the case and since it is impractical to monitor all possible populations, the Surveillance Tree methodology provides an efficient but flexible method of detection. It can be thought of as a forward selection multivariate scan plan. The method’s efficiencies come from providing a targeted method for finding the best level of data aggregation and so avoiding the aggregation of subspaces where no outbreak is occurring. This level of aggregation in the multivariate data is also able to be determined with manageable false alarm rates. Furthermore, the use of this computationally feasible, multivariate, partitioned surveillance method takes health care managers one step closer to acting on the hotspot by providing information about its diagnosis.

## Competing interests

The authors declare that they have no competing interests.

## Authors’ contributions

SB performed all data preparation, coding and analysis and drafted the manuscript. RS conceived of the study, and advised on all stages of its design and execution and helped to draft the manuscript. Both authors read and approved the final manuscript.

## Pre-publication history

The pre-publication history for this paper can be accessed here:

http://www.biomedcentral.com/1472-6947/13/132/prepub
